# Supplementary material for: The FDA-Approved Anti-Asthma Medicine Ciclesonide Inhibits Lung Cancer Stem Cells through Hedgehog Signaling-Mediated SOX2 Regulation
Source: Int J Mol Sci. 2020 Feb 4;21(3):1014. doi: 10.3390/ijms21031014 (PMC7038186; doi:10.3390/ijms21031014)
Supplement: Supplementary file 1 [file ijms-21-01014-s001.pdf]

Table S1. Specific real-time (RT)-qPCR primer sequences containing *GLI1, GLI2, GLI3, c-Myc, Snail, Nanog, Sox2,* and *β-actin* genes.

Genes	Primers
GLI1	Forward: 5'-CAGGGAAGAGAGCAGACTGAC-3'<br>Reverse: 5'-CAGGAGGATTGTGCTCCA-3'
GLI2	Forward: 5'-ATCCCCGCTTGGACTGAC-3'<br>Reverse: 5'-ACCTCGGCCTCCTGCTTA-3'
GLI3	Forward: 5'-GGCCTCCAGTACCACTTCAA-3'<br>Reverse: 5'-CTGAGACCCTGCACACTCTG-3'
c-Myc	Forward: 5'-CCTGGTGCTCCATGAGGAGAC-3'<br>Reverse: 5'-CAGACTCTGACCTTTTGCCAGG-3'
Snail	Forward: 5'-GCTGCAGGACTCTAATCCAGA-3'<br>Reverse: 5'-ATCTCCGGAGGTGGGATG-3'
Nanog	Forward: 5′-ATGCCTCACACGGAGACTGT-3′<br>Reverse: 5′-AAGTGGGTTGTTTGCCTTTG-3′
SOX2	Forward: 5′-TTGCTGCCTCTTTAAGACTAGGA-3′<br>Reverse: 5′-CTGGGGCTCAAACTTCTCTC-3′
β-actin	Forward: 5′-TGTTACCAACTGGGACGACA-3′<br>Reverse: 5′-GGGGTGTTGAAGGTCTCAAA-3′

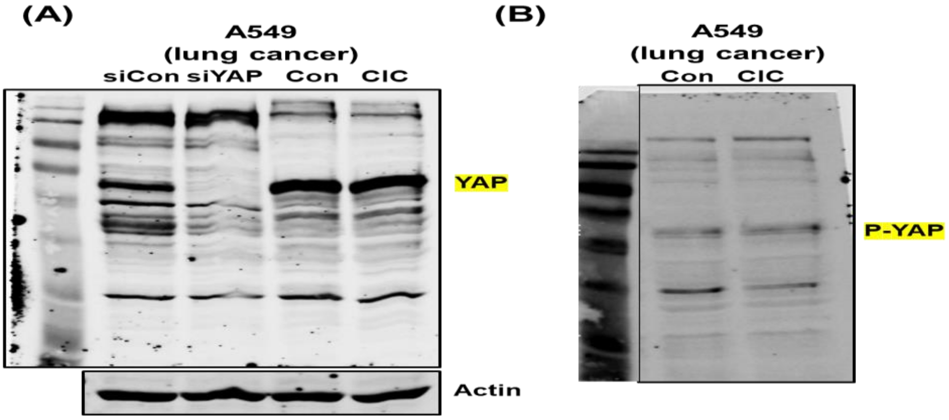

Figure S1. The effect of ciclesonide on expression levels of YAP1 and phosphoYAP1 in lung cancer (A549 cells). (A) Western blot analysis of the YAP protein under ciclesonide (CIC) and the knock-down of YAP using siRNA in A549 cells. (B) Western blot analysis of the pYAP protein under CIC in A549 cells.

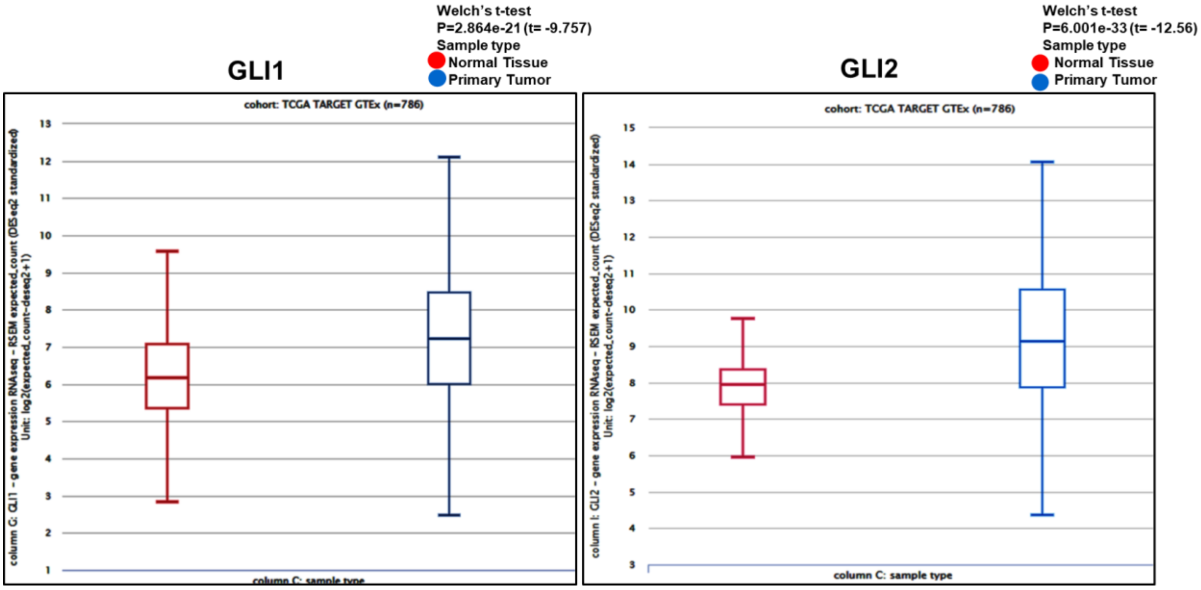

Figure S2. GLI1 and GLI2 expression in primary tumor tissue and normal tissue using the Cancer Genome Atlas (TCGA) lung cancer cohort.
